# Toll‐like receptor 4 polymorphisms in Saudi population with cardiovascular diseases

**DOI:** 10.1002/mgg3.852

**Published:** 2019-07-21

**Authors:** Abdelhabib Semlali, Mikhlid Al Mutairi, Ibrahim Oqla Alanazi, Hasan Awad Aljohi, Narasimha Reddy Parine, Abdullah Alhadheq, Abdulaziz A. Al‐Jafari, Abdulelah F. Mobeirek, Abdullah Al Amri, Jilani P. Shaik, Fatima‐zohra Filali, Mohammad Alanazi

**Affiliations:** ^1^ Groupe de Recherche en Écologie Buccale, Faculté de Médecine Dentaire Université Laval Québec QC Canada; ^2^ Genome Research Chair, Department of Biochemistry College of Science King Saud University Riyadh Kingdom of Saudi Arabia; ^3^ Zoology Department College of Science King Saud University Riyadh Kingdom of Saudi Arabia; ^4^ National Center for Genomics Research (NCGR), King Abdulaziz City for Science and Technology Riyadh Saudi Arabia; ^5^ Department of Biochemistry, Faculty of Science King Saud University Riyadh Kingdom of Saudi Arabia; ^6^ Cardiac Sciences Department, Faculty of Medicine King Saud University Riyadh Kingdom of Saudi Arabia; ^7^ Centre Hospitalier Provincial (CHP) de Taounate Taounate Maroc

**Keywords:** cardiovascular disease, innate immune system, single‐nucleotide polymorphism, Toll‐Like Receptor 4

## Abstract

**Background:**

Toll‐like receptors play a substantial role in innate immunity and the effects of *TLR4* genetic variants on cardiovascular diseases are still largely unknown. Therefore, we aimed to investigate the effects of *TLR4* polymorphisms on cardiovascular diseases risk in the Saudi population.

**Methods:**

Three tag single‐nucleotide polymorphisms (rs2770150, rs10759931, and rs4986790) in TLR4 were studied on 222 unrelated patients with cardiovascular diseases and 190 healthy volunteers.

**Results:**

We found that, in patients over 60 years old, the frequency of the TT genotype in rs2770150 and the variant allele G in rs10759931 were higher compared to the control group. Based on gender, the genotype frequency of rs2770150 increases the risk for cardiovascular diseases in female patients by 3.6‐fold. The allele frequency for the G allele of rs10759931 increased the risk for CVDs in male patients by more than 1.5‐fold. Furthermore, the genotype frequency of rs2770150 had a significant association with cardiovascular diseases in patients without hypertension and G allele of rs10759931 significantly increased the risk of cardiovascular diseases in patients that smoked. After Bonferroni correction only patients without hypertension showed significant risk of CVD with rs2770150.

**Conclusion:**

A deeper understanding of the genetic variability of TLR4 will enable us to better identification of biomarkers for early detection and prognosis, and also enhance the decision‐making process of treatments for cardiovascular diseases.

## Graphical abstract

The genotype frequency of rs2770150 had a significantly association with Cardio Vascular diseases in Saudi patients.

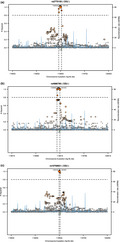

## 1 INTRODUCTION

Cardiovascular diseases (CVDs) are one of the most common diseases that result in mortality in the western world, which represents 31% of deaths worldwide. Various factors such as genetics, tobacco use, diet, exercise, and metabolism have been identified and studied in the context of CVDs; however, the complete picture for the pathogenesis of CVDs remain unclear (Gibson et al., 2004). It is crucial to understand the cellular and molecular mechanisms and genetic risk factors involved in CVDs. Considerable evidence have indicated that the deregulation of the innate immune system may increase the risk for CVDs (Abboud, Harwani, & Chapleau, 2012; Coutinho & Chapman, 2011; Mann, 2011; Yang et al., 2008). However, it has been shown that the heart possesses a powerful innate immune system that is able to delimit tissue injuries, as well as orchestrate homoeostatic responses within the heart (Mann, 2011). Furthermore, this innate immune system is at least in part mediated by a family of Toll‐like receptors (TLRs), and is considered the first line of defense against foreign pathogens by responding to signals that are induced by microbial infections or endogenous in origin (Lin, Freedman, & Beaulieu, 2009). CVDs are a complex disorder involving several pathophysiological processes such as the activation of TLRs, which are germline‐encoded receptors that recognize exogenous as well as tissue‐derived molecular signals that promote inflammation. Additionally, TLRs are found in various tissues and cell types including cardiomyocytes, vascular smooth muscle, and endothelial cells (Spirig, Tsui, & Shaw, 2012). Ten TLRs have been characterized in humans, and each one of them recognizes ligands that create unique responses depending on the context. TLRs could act as key regulators involved in immune system activation, cardiovascular disease progression, and chronic inflammation (Spirig et al., 2012) and are one of the best characterized pathogen recognition receptors. This receptor consists of an extracellular leucine‐rich repeat domain, a transmembrane domain, and an intracellular Toll/interleukin‐1 (IL‐1) receptor (TIR) domain. The signaling pathway of TLR4 is vital because it is capable of recruiting all four known TIR‐containing adaptor molecules (Vogel, Fitzgerald, & Fenton, 2003). TLR4 signaling can be initiated by the binding of pathogen‐associated molecular patterns and also by binding putative endogenous ligands created during inflammation and tissue injury (Noreen et al., 2012). Exogenous ligands of this receptor include lipopolysaccharides (LPSs) of Gram‐negative bacteria (Takeda & Akira, 2005), soluble components of Mycobacterium tuberculosis, and manna's from fungal pathogens, whereas endogenous ligands include a number of heat shock proteins and fibronectin (Noreen et al., 2012). The binding of exogenous or endogenous ligands to TLR4 initiates the induction of a signal transduction cascade. TLR4 also cooperates with myeloid differentiation protein‐2, LPS‐binding protein, and cluster of differentiation 14 to mediate the innate immune response to bacterial LPS (Zou et al., 2015). Two pathways have been identified for TLR4 signaling. The first pathway is dependent on myeloid differentiation primary response gene 88 (MyD88). Activation of this pathway leads to the activation of the mitogen‐activated protein kinase cascade and nuclear factor kappa B, which induces the expression of proinflammatory cytokines. In contrast, the initiation of the MyD88‐independent pathway results in the activation of interferon (IFN) regulatory factor 3, IFN‐inducible genes, and the expression of IFN‐β (Lu, Yeh, & Ohashi, 2008; Werling & Jungi, 2003). TLR signaling in vivo can be impaired due to the presence of single‐nucleotide polymorphisms (SNPs) within the TLR genes, thus altering the susceptibility to viral infections, fungal and bacterial allergic diseases (Schröder & Schumann, 2005), inflammation, and cancers (Medvedev, 2013). Previous studies of SNPs in TLRs demonstrated that TLRs are involved in the development and progression of diseases such as atherosclerosis, cardiac dysfunction in sepsis, and congestive heart failure (Frantz, Ertl, & Bauersachs, 2007). A number of synonymous and nonsynonymous SNPs have been identified in the promoter and coding regions of *TLR4*. SNPs in these regions are associated with infectious and inflammatory diseases such as Gram‐negative infections, sepsis, RSV bronchiolitis, and inflammatory bowel disease (Medvedev, 2013). Likewise, SNPs in these regions of *TLR4* have also been linked to an increased incidence of several diseases such as colon (Semlali et al., 2016), breast (Theodoropoulos et al., 2012), prostate, cervical cancer (2011), and cardiovascular disease (Eisler et al., 2011; Liu et al., 2015). Previous studies have also shown a possible association between the TLR4‐Asp299Gly polymorphism and cardiovascular diseases. Kolek et al. (2004) showed that the TLR4‐Asp299Gly polymorphism was associated with reductions in vascular inflammation and angiographic coronary artery disease (CAD; Kolek et al., 2004). Similarly, two studies by Ameziane et al. and Balisteri et al. confirmed an association between the TLR4‐Asp299Gly polymorphism with a lower risk for acute coronary events independent of known coronary risk factors (Ameziane et al., 2003; Balistreri et al., 2004). In contrast, others have shown that there is no association between the TLR4‐Asp299Gly polymorphism with coronary artery stenosis (Yang, Holloway, & Ye, 2003) or cerebral ischemia (Guven et al., 2015; Reismann et al., 2004). The rs4986790 SNP in *TLR4* has been shown to have little evidence of association between the Asp299Gly gene polymorphism and risks for incident myocardial infarction (Zee, Hegener, Gould, & Ridker, 2005) whereas the rs2770150 and rs10759931 SNPs have not been associated with cardiovascular disease. Therefore, the current study is the first to investigate the effects of three *TLR4* SNPs (rs4986790, rs2770150, and rs10759931) on the risk for CVDs in the Saudi population.

## 2 MATERIALS AND METHODS

### 2.1 Patient population and ethics statement

The study cohort included 416 individuals, which consisted of 226 patients clinically hospitalized for CVDs at King Khalid University Hospital (KKUH) in Riyadh, Kingdom of Saudi Arabia (KSA), and a control group of 190 healthy age‐ and sex‐matched blood donors. Their ethnicities were verified, since parents and grandparents of the patients and controls were born in Saudi Arabia. The study protocol was reviewed and approved by the local ethic committee from KKUH, and all study patients gave their written informed consent. A baseline questionnaire was performed to gather information on age, demographics, sex, clinical features (e.g., disease duration), and behavioral (e.g., smoking) factors. Hypertension and hypotension events and pharmacological treatments were also collected in the questionnaire and then confirmed by medical records, and all of the clinical data are summarized in Table 1. All Saudi patients included in this study did not suffer from any other diseases or other known pathologies, and have no family history of CVDs. This study is conform to the principles outlined in the Declaration of Helsinki. The mean age of the study population was 61.77 ± 10.33 years for the patients with CVDs and 59.35 ± 6.37 years for the healthy controls (Table 1). Approximately 4–5 ml of blood were collected from each volunteer and immediately stored in EDTA tubes at −80°C until they were used for genomic DNA extraction.

**Table 1:** Demographic characteristics of the cardiovascular disease (CVD) patients and control samples

Characteristic	CVD group *n* = 226 (%)	Controls *n* = 190 (%)
Age, years	61.77 ± 10.33	59.35 ± 6.37
Gender		
Male	154 (68.14%)	100 (52.63%)
Female	72 (31.86%)	90 (47.37%)
FBS, mmol/L	8.06 ± 3.4	—
TG, mmol/L	1.86 ± 1.19	—
TC, mmol/L	4.16 ± 0.98	—
HDL‐c, mmol/L	1.15 ± 0.94	—
LDL‐c, mmol/L	2.37 ± 0.84	—
Smoking	90 (39.82%)	86 (45.26%)
Nonsmoker	136 (60,18%)	104 (54.74%)
Hyper and hypotension events	166 (73,45%) 60 (92.55%)	—
Pharmacological treatments	Yes = 155 (68.58%) No = 71 (31.42%)	No treatments

### 2.2 DNA extraction

Blood DNA extraction was performed as previously described (Kohailan et al., 2016; Semlali, Parine, et al., 2017; Semlali et al., 2016). Genomic DNA was extracted from the blood samples with the QIAamp DNA Mini Kit (Qiagen) based on the manufacturer's instructions. For each blood sample, 4 ml of the sample was equilibrated at room temperature, mixed with protease and lysis buffer, and incubated at 56°C for 10 min. An equal volume of 70% absolute ethanol was added to each sample, mixed, and then passed through the column via centrifugation. The column was then washed with 2 ml of buffer AW1 and then 2 ml of buffer AW2 to ensure complete drying. Finally, the extracted DNA was eluted with 100 μl of the elution buffer (AE). A NanoDrop spectrometer was used to estimate the DNA concentration and purity using the A260/A280 ratio.

### 2.3 Choice of SNPs

Recently, our studies and others have identified three SNPs in *TLR4*, including rs2770150, rs10759931, and rs4986790 that were associated with colon and breast cancer in the Saudi population (Semlali, Jalouli, et al., 2017; Semlali et al., 2016). These SNPs were selected based on the HapMap database (http://www.hapmap.org) due to their functional properties. Furthermore, these SNPs are located in the regulatory region of *TLR4* in the 5′UTR, promoter region, and exon‐coding region, respectively (Table S1).

### 2.4 Genotyping assay

Genotyping for the *TLR4* SNPs was performed in a 96‐well plate by using a TaqMan allelic discrimination assay as previously described (Alanazi et al., 2013; Kohailan et al., 2016; Semlali, Parine, et al., 2017; Semlali et al., 2016) and by using the TaqMan^®^ SNP Genotyping Assay from Applied Biosystems according to the manufacturer's protocol (Semlali, Parine, et al., 2017; Semlali et al., 2016). Briefly, the amplification was performed in a final reaction volume of 10 μl containing 20 ng of genomic DNA, 5.6 μl of 2X Universal Genotyping Master Mix, and 200 nM of each primer. The allelic discrimination was performed on the ABI 7500 real‐time PCR machine (Applied Biosystems). The following amplification cycle conditions were used: 60°C for 30 s and 95°C for 10 min, followed by 40 cycles of 95°C for 15 s, 60°C for 1 min, and a postread stage of 60°C for 30 s. To verify the genotyping procedures, 5% of the samples selected were genotyped in duplicates a repeat analysis was performed for quality control.

### 2.5 Statistical analysis

Genotype frequencies were tested according to the Hardy–Weinberg equilibrium exact test. Significant differences in frequencies between the controls were calculated using the *χ* ^2^ test with Yates corrections. Odds ratios (ORs) and 95% confidence intervals (CIs) were calculated with the Fisher's exact test (two‐tailed) to evaluate the disease risk. The Statistical Package for the Social Sciences 16.0 software for Windows was used for the statistical analysis. We considered *p* < .05 as significant. Finally, Bonferroni's correction was applied for multiple comparisons.

## 3 RESULTS

### 3.1 Analysis of clinical characteristics and identification of a combined CVD risk genotype related to the three *TLR4* SNPs in Saudi patients

The detailed clinical characteristics of the 226 cases of CVDs and 190 matched controls, including age, sex, ethnic, family history, smoking habits, and pharmacological treatments are shown in Table 1. Of the patients with CVDs, 68.14% were males and 31.56% were females, whereas 52.63% were males and 47.37% were females in the control group. The ratio of males to females was not considerably dissimilar between the cases and controls. It should be noted that the ethnic distribution was 100% Saudi Arabia for all study subjects. The mean age of the study cohort was 61.77 ± 10.33 years and 59.35 ± 6.37 years for patients with CVDs and healthy subjects, respectively. The difference in the mean age between the two groups was not significant (*p* > .05).

To investigate the key role of *TLR4* polymorphisms in the pathophysiology of CVDs, we compared the allele frequencies of three selected SNPs between patients with CVD and healthy controls. The distribution of alleles and genotypes with OR and significances as well as the association analysis are reported in Table 2. Hardy–Weinberg equilibrium was used to measure the genotype frequencies in all SNPs including rs2770150 T/C (or C/T), rs4986790 A/G, and rs1075993 A/G. The homozygous ancestral allele was used as a reference to determine the possible risk of acquiring CVDs associated with the other two genotypes. Of the three SNPs in *TLR4* that were analyzed, no statistically significant associations between these SNPs and patients with CVDs were found (Table 2). However, the distribution of genotype frequencies of rs2770150, rs10759931, and rs4986790 was not statistically significant between the cases of CVD and controls (*p* = .70881, *p* = .35100, and *p* = .78247, respectively). The TT genotype of rs2770150 was found at a lower frequency in cases of CVDs (0.17) compared to controls (0.11) as well as a 1.6‐fold increased risk against cardiovascular diseases (Table 2). For rs4986790, the distribution of genotype and allele frequencies between the patients with CVDs and healthy controls were similar (Table 2), which was similar to that of the GG and AG genotypes of rs4986790.

**Table 2:** Genotype frequencies of TLR‐4 gene polymorphism in cardiovascular and controls

Gene	SNP ID	Genotype	Cardiovascular	Controls	OR	95% CI	χ^2^‐value	*p* *‐value
TLR‐4	rs2770150	CC	100 (0.45)	88 (0.47)	Ref			
		CT	85 (0.38)	81 (0.42)	0.923	0.608–1.403	0.14	.70881
		TT	37 (0.17)	20 (0.11)	1.628	0.880–3.011	2.44	.11845
		CT + TT	122 (0.55)	101 (0.53)	1.063	0.720–1.569	0.09	.75852
		C	285 (0.64)	257 (0.68)	Ref			
		T	159 (0.36)	121 (0.32)	1.185	1.31	1.31	.25188
TLR‐4	rs4986790	AA	187 (0.85)	166 (0.87)	Ref			
		AG	30 (0.14)	20 (0.11)	1.332	0.728–2.434	0.87	.35100
		GG	0 (0.00)	1 (0.01)	0.296	0.012–7.316	1.12	.28929
		AG + GG	30 (0.14)	21 (0.12)	1.268	0.699–2.300	0.61	.43359
		A	404 (0.92)	352 (0.92)	Ref			
		G	30 (0.07)	22 (0.06)	1.188	0.673–2.098	0.35	.55186
TLR‐4	rs10759931	AA	13 (0.06)	15 (0.08)	Ref			
		AG	69 (0.31)	71 (0.38)	1.121	0.497–2.529	0.08	.78247
		GG	137 (0.63)	100 (0.54)	1.581	0.720–3.470	1.32	.25066
		AG + GG	206 (0.94)	171 (0.92)	1.390	0.644–3.002	0.71	.40011
		A	95 (0.22)	101 (0.27)	Ref			
		G	343 (0.78)	271 (0.73)	1.346	0.975–1.857	3.27	.07053

* *p* < .05.

### 3.2 Association between *TLR4* SNPs and the risk for developing CVDs based on age at disease diagnosis and gender

We investigated whether there was an association between the *TLR4* SNPs rs2770150, rs10759931, and rs4986790 with the age at which CVDs were diagnosed. The patients were stratified into two groups based on the median age at the time of disease diagnosis. The first group consisted of patients less than 60 years of age (*n* = 127) and the second group consisted of patients older than 60 years of age (*n* = 95). The genotype and allele frequencies were compared to the age‐matched controls and the results are reported in (Table 3).

**Table 3:** Genotype frequencies of TLR‐4 gene polymorphism in cardiovascular and controls

Gene	SNP ID	Genotype	Cardiovascular	Controls	OR	95% CI	χ^2^‐value	*p* *‐value
(A) Age below 60 years								
TLR‐4	rs2770150	CC	64 (0.50)	47 (0.49)	Ref			
		CT	49 (39)	40 (0.42)	0.900	0.513–1.579	0.14	.71228
		TT	14 (0.11)	9 (0.09)	1.142	0.456–2.861	0.08	.77622
		CT + TT	63 (0.50)	49 (0.51)	0.944	0.556–1.604	0.05	.83190
		C		134 (0.70)	Ref			
		T	77 (0.30)	58 (0.30)	1.005	0.668–1.511	0.00	.98064
TLR‐4	rs4986790	AA	104 (0.85)	88 (0.91)	Ref			
		AG	19 (0.15)	8 (0.08)	2.010	0.839–4.813	2.52	.11210
		GG	0 (0.00)	0 (0.00)	0.847	0.017–43.119	0.001	1.00000
		AG + GG	19 (0.15)	8 (0.08)	2.010	0.839–4.813	2.52	.11210
		A	227 (0.92)	184 (0.95)	Ref			
		G	19 (0.08)	8 (0.04)	1.925	0.824–4.498	2.36	.12460
TLR‐4	rs10759931	AA	7 (0.06)	5 (0.05)	Ref			
		AG	47 (0.38)	39 (0.41)	0.861	0.253–2.926	0.06	.81015
		GG	71 (0.56)	50 (0.53)	1.014	0.304–3.379	0.00	.98157
		AG + GG	118 (0.94)	89 (0.94)	0.947	0.291–3.082	0.01	.92797
		A	61 (0.24)	49 (0.26)	Ref			
		G	189 (0.76)	139 (0.74)	1.092	0.707–1.688	0.16	.69105
(B) Age above 60 years								
TLR‐4	rs2770150	CC	36 (0.38)	41 (0.44)	Ref			
		CT	36 (0.38)	41 (0.44)	1.000	0.531–1.883	0.00	1.000
		TT	23 (0.24)	11 (0.12)	2.381	1.021–5.552	4.13	.04201^ns^
		CT + TT	59 (0.62)	52 (0.56)	1.292	0.722–2.314	0.74	.38809
		C	108 (0.56)	123 (0.66)	Ref			
		T	82 (0.43)	63 (0.34)	1.482	0.976–2.251	3.42	.06435
TLR‐4	rs4986790	AA	83 (0.88)	78 (0.85)	Ref			
		AG	11 (0.11)	12 (0.14)	0.861	0.359–2.066	0.11	.73804
		GG	0 (0.00)	1 (0.01)	0.313	0.013–7.807	1.06	.30386
		AG + GG	11 (0.11)	13 (0.15)	0.795	0.336–1.880	0.27	.60108
		A	177 (0.94)	168 (0.92)	Ref			
		G	11 (0.06)	14 (0.08)	0.746	0.329–1.689	0.50	.48055
TLR‐4	rs10759931	AA	6 (0.06)	10 (0.11)	Ref			
		AG	22 (0.23)	32 (0.35)	1.146	0.363–3.613	0.05	.81622
		GG	66 (0.70)	50 (0.54)	2.200	0.750–6.457	2.13	.14410
		AG + GG	88 (0.93)	82 (0.89)	1.789	0.622–5.141	1.19	.27525
		A	34 (0.18)	52 (0.28)	Ref			
		G	154 (0.82)	132 (0.72)	1.784	1.092–2.915	5.42	.01994^ns^

ns = After Bonferroni correction *p* > .05.

* *p* < .05.

Individuals with the TT genotype compared to the CC genotype in rs2770150 were at a significantly higher risk (2.38‐fold) of developing CVDs, but only in patients over 60 years of age (Table 3A,B). The TT genotype frequency in rs2770150 was higher in patients with CVDs (24%) compared to the control (12%; OR = 2.381, 95% CI = 1.021–5.552, and *p* = .04201). Additionally, the genotype and allele frequencies between the patients with CVDs and the healthy controls were almost similar for rs4986790 in both groups. For rs10759931, our analysis showed that the allele frequencies of the G allele increases the risk for developing CVD in patient more than 60 years old by 1.7‐fold, where the G allele frequency was 82% in patients with CVDs compared to 72% in healthy controls (OR = 1.782, 95% CI = 1.092–2.915, and *p* = .01994; Table 3B). This result suggested that the minor G allele significantly increases the risk for CVDs (Table 3A,B). After Bonferroni correction variable age did not showed any significant risk of CVD with any SNP.

The prevalence of genotype and allele frequencies of the three SNPs in *TLR4* was then examined with regards to gender (Table 4). In the male population, individuals with the G allele of rs10759931 had a 1.5‐fold increased risk for developing CVDs (OR = 1.0559, 95% CI = 1.031–2.358, *p* = 0.03454). However, in the female population, the G allele frequency was similar between patients with CVDs (78%) and health controls (76%; Table 4A,B). This suggests that the minor G allele confers a significant higher risk for developing CVDs in the Saudi population. Furthermore, the GG genotype was not associated with the gender in patients with CVDs (Table 4A). Conversely, individuals with the TT genotype of rs2770150 have a 3.6‐fold higher risk for developing CVDs when compared to those with the CC genotype in the female population only (males: OR = 0.996, 95% CI = 0.469–2.092, *p* = .97970; females: OR = 3.639, 95% CI = 1.167–11.35, *p* = .02061). Additionally, based on the genotype and allele frequencies, rs4986790 did not show any association with the risk for CVD susceptibility in both groups of the overall study population (Table 4A,B). After Bonferroni correction variable gender did not showed any significant risk of CVD with any SNP.

**Table 4:** Genotype frequencies of TLR‐4 gene polymorphism in cardiovascular and controls

Gene	SNP ID	Genotype	Cardiovascular	Controls	OR	95% CI	χ^2^‐value	*p* *‐value
(A) Based on gender (male)								
TLR‐4	rs2770150	CC	69 (0.45)	41 (0.41)	Ref			
		CT	58 (0.38)	44 (0.44)	0.783	0.452–1.358	0.76	.38401
		TT	25 (0.17)	15 (0.15)	0.990	0.469–2.092	0.000	.97970
		CT + TT	83 (0.55)	59 (0.59)	0.836	0.502–1.393	0.47	.49133
		C	196 (0.64)	126 (0.63)	Ref			
		T	108 (0.36)	74 (0.37)	0.938	0.647–1.360	0.11	.73613
TLR‐4	rs4986790	AA	129 (0.87)	89 (0.90)	Ref			
		AG	19 (0.13)	9 (0.09)	1.457	0.630–3.366	0.78	.37698
		GG	0 (0.00)	1 (0.01)	0.230	0.009–5.719	1.44	.23015
		AG + GG	19 (0.13)	10 (0.10)	1.311	0.582–2.952	0.43	.51259
		A	277 (0.94)	187 (0.94)	Ref			
		G	19 (0.06)	11 (0.05)	1.166	0.542–2.507	0.16	.69377
TLR‐4	rs10759931	AA	11 (0.07)	10 (0.10)	Ref			
		AG	42 (0.28)	38 (0.40)	1.005	0.384–2.630	0.00	.99224
		GG	96 (0.64)	49 (0.50)	1.781	0.708–4.482	1.53	.21603
		AG + GG	138 (0.92)	87 (0.90)	1.442	0.588–3.538	0.64	.42207
		A	64 (0.21)	58 (0.30)	Ref			
		G	234 (0.79)	136 (0.70)	1.559	1.031–2.358	4.47	.03454^ns^
(B) Based on gender (female)								
TLR‐4	rs2770150	CC	31 (0.44)	47 (0.53)	Ref			
		CT	27 (0.39)	37 (0.42)	1.106	0.565–2.167	0.09	.76815
		TT	12 (0.17)	5 (0.05)	3.639	1.167–11.350	5.36	.02061^ns^
		CT + TT	39 (0.56)	42 (0.47)	1.408	0.751–2.641	1.14	.28587
		C	89 (0.64)	131 (0.74)	Ref			
		T	51 (0.36)	47 (0.26)	1.597	0.989–2.579	3.69	.05463
TLR‐4	rs4986790	AA	58 (0.84)	77 (0.86)	Ref			
		AG	11 (0.16)	11 (0.13)	1.328	0.538–3.274	0.38	.53745
		GG	0 (0.00)	0 (0.00)	1.325	0.026–67.752	nan	1.0000
		AG + GG	11 (0.16)	11 (0.13)	1.328	0.538–3.274	0.38	.53745
		A	127 (0.92)	165 (0.94)	Ref			
		G	11 (0.08)	11 (0.06)	1.299	0.546–3.092	0.35	.55319
TLR‐4	rs10759931	AA	2 (0.02)	5 (0.06)	Ref			
		AG	27 (0.39)	33 (0.37)	2.045	0.367–11.387	0.69	.40644
		GG	41 (0.59)	51 (0.57)	2.010	0.371–10.898	0.68	.41053
		AG + GG	68 (0.98)	84 (0.94)	2.024	0.381–10.758	0.71	.39957
		A	31 (0.22)	43 (0.24)	Ref			
		G	109 (0.78)	135 (0.76)	1.120	0.662–1.896	0.18	.67301

ns = After Bonferroni correction *p* > .05.

* *p* < .05.

### 3.3 Association between *TLR4* SNPs and risk of developing cardiovascular diseases in the Saudi population based on hypertension events and behavioral factors at disease diagnosis

We investigated the risk for predisposition to CVDs in association with the three SNPs in *TLR4* in the Saudi population based on hypertension events and smoking conditions. The comparisons of genotype and allelic frequencies in the CVD group and the healthy populations are presented in Tables 5 and 6. Of the three SNPs in *TLR* that were analyzed, a statistically significant association with the risk for CVDs was observed for rs2770150 only in patients with CVDs without hypertension. The TT genotype frequency was 26% in patients with CVDs without hypertension and 11% in healthy controls. Nonetheless, it was noted that the genotype and allele frequencies were not affected by hypertension events in cases of CVDs. The genotypes of rs4986790 and rs10759931 did not show any predisposition to CVDs in patients with regards to hypertension (Table 5A,B).

**Table 5:** Genotype frequencies of TLR‐4 gene polymorphism in cardiovascular and controls based on hyper‐hypotension events

Gene	SNP ID	Genotype	Cardiovascular	Controls	OR	95% CI	χ^2^‐value	*p* *‐value
(A) hypertension events								
TLR‐4	rs2770150	CC	75 (0.46)	88 (0.47)	Ref			
		CT	66 (0.41)	81 (0.42)	0.956	0.611–1.496	0.04	.84403
		TT	21 (0.13)	20 (0.11)	1.232	0.621–2.445	0.36	.55043
		CT + TT	87 (0.54)	101 (0.53)	1.011	0.664–1.539	0.00	.96049
		C	216 (0.67)	257 (0.68)	Ref			
		T	108 (0.33)	121 (0.32)	1.062	0.774–1.457	0.14	.70940
TLR‐4	rs4986790	AA	136 (0.86)	166 (0.87)	Ref			
		AG	23 (0.14)	20 (0.11)	1.404	0.740–2.664	1.08	.29802
		GG	0 (0.00)	1 (0.01)	0.407	0.016–10.061	0.82	.36604
		AG + GG	23 (0.14)	21 (0.12)	1.337	0.709–2.519	0.81	.36799
		A	295 (0.93)	352 (0.92)	Ref			
		G	23 (0.07)	22 (0.06)	1.247	0.681–2.284	0.52	.47279
TLR‐4	rs10759931	AA	8 (0.05)	15 (0.08)	Ref			
		AG	52 (0.33)	71 (0.38)	1.373	0.542–3.479	0.45	.50257
		GG	99 (0.62)	100 (0.54)	1.856	0.753–4.575	1.85	.17383
		AG + GG	151 (0.95)	171 (0.92)	1.656	0.683–4.014	1.27	.26026
		A	68 (0.21)	101 (0.27)	Ref			
		G	250 (0.79)	271 (0.73)	1.370	0.963–1.949	3.08	.07911
(B) No hypertension events								
TLR‐4	rs2770150	CC	25 (0.42)	88 (0.47)	Ref			
		CT	19 (0.32)	81 (0.42)	0.826	0.423–1.611	0.32	.57410
		TT	16 (0.26)	20 (0.11)	2.816	1.274–6.226	6.82	.00902*
		CT + TT	35 (0.58)	101 (0.53)	1.220	0.678–2.195	0.44	.50707
		C	69 (0.58)	257 (0.68)	Ref			
		T	51 (0.42)	121 (0.32)	1.570	1.030–2.393	4.43	.03526^ns^
TLR‐4	rs4986790	AA	51 (0.88)	166 (0.87)	Ref			
		AG	7 (0.12)	20 (0.11)	1.139	0.456–2.848	0.08	.78025
		GG	0 (0.00)	1 (0.01)	1.078	0.043–26.860	0.31	.57965
		AG + GG	7 (0.12)	21 (0.12)	1.085	0.436–2.698	0.03	.86072
		A	109 (0.94)	352 (0.92)	Ref			
		G	7 (0.06)	22 (0.06)	1.028	0.427–2.470	0.00	.95163
TLR‐4	rs10759931	AA	5 (0.08)	15 (0.08)	Ref			
		AG	17 (0.28)	71 (0.38)	0.718	0.229–2.251	0.32	.56902
		GG	38 (0.63)	100 (0.54)	1.140	0.388–3.353	0.06	.81175
		AG + GG	55 (0.91)	171 (0.92)	0.965	0.335–2.776	0.00	.94718
		A	27 (0.23)	101 (0.27)	Ref			
		G	93 (0.77)	271 (0.73)	1.284	0.790–2.086	1.02	.31264

ns = After Bonferroni correction *p* > .05.

* After Bonferroni correction *p* < .05.

**Table 6:** Genotype frequencies of TLR‐4 gene polymorphism cardiovascular and controls depending on behavior factor

Gene	SNP ID	Genotype	Cardiovascular	Controls	OR	95% CI	χ^2^‐value	*p* *‐value
(A) In smoker patients								
TLR‐4	rs2770150	CC	42 (0.47)	88 (0.47)	Ref			
		CT	33 (0.37)	81 (0.42)	0.854	0.494–1.475	0.32	.57032
		TT	14 (0.16)	20 (0.11)	1.467	0.675–3.185	0.94	.33158
		CT + TT	47 (0.53)	101 (0.53)	0.975	0.589–1.615	0.01	.92174
		C	117 (0.66)	257 (0.68)	Ref			
		T	61 (0.34)	121 (0.32)	1.107	0.759–1.615	0.28	.59638
TLR‐4	rs4986790	AA	73 (0.85)	166 (0.87)	Ref			
		AG	13 (0.15)	20 (0.11)	1.478	0.698–3.131	1.05	.30541
		GG	0 (0.00)	1 (0.01)	0.755	0.030–18.755	0.44	.50763
		AG + GG	13 (0.15)	21 (0.12)	1.408	0.669–2.964	0.82	.36634
		A	159 (0.92)	352 (0.92)	Ref			
		G	13 (0.08)	22 (0.06)	0.764	0.376–1.556	0.55	.45771
TLR‐4	rs10759931	AA	3 (0.03)	15 (0.08)	Ref			
		AG	28 (0.32)	71 (0.38)	1.972	0.530–7.341	1.06	.30430
		GG	57 (0.65)	100 (0.54)	2.850	0.791–10.266	2.76	.09638
		AG + GG	85 (0.97)	171 (0.92)	2.485	0.700–8.820	2.11	.14640
		A	34 (0.19)	101 (0.27)	Ref			
		G	142 (0.81)	271 (0.73)	1.557	1.004–2.413	3.95	.04694^ns^
(B) No smoker								
TLR‐4	rs2770150	CC	58 (0.44)	88 (0.47)	Ref			
		CT	52 (0.39)	81 (0.42)	0.974	0.602–1.575	0.01	.91459
		TT	23 (0.17)	20 (0.11)	1.745	0.880–3.461	2.57	.10898
		CT + TT	75 (0.56)	101 (0.53)	1.127	0.721–1.760	0.27	.60035
		C	168 (0.63)	257 (0.68)	Ref			
		T	98 (0.37)	121 (0.32)	1.239	0.891–1.723	1.62	.20253
TLR‐4	rs4986790	AA	114 (0.88)	166 (0.87)	Ref			
		AG	17 (0.13)	20 (0.11)	1.238	0.621–2.465	0.37	.54360
		GG	0 (0.00)	1 (0.01)	0.485	0.020–12.004	0.69	.40785
		AG + GG	17 (0.13)	21 (0.12)	1.179	0.596–2.332	0.22	.63639
		A	245 (0.94)	352 (0.92)	Ref			
		G	17 (0.06)	22 (0.06)	1.110	0.578–2.134	0.10	.75381
TLR‐4	rs10759931	AA	10 (0.08)	15 (0.08)	Ref			
		AG	41 (0.31)	71 (0.38)	0.866	0.357–2.104	0.10	.75102
		GG	80 (0.61)	100 (0.54)	1.200	0.512–2.814	0.18	.67478
		AG + GG	121 (0.92)	171 (0.92)	1.061	0.461–2.442	0.02	.88852
		A	61 (0.23)	101 (0.27)	Ref			
		G	201 (0.77)	271 (0.73)	1.228	0.851–1.772	1.21	.27151

ns = After Bonferroni correction *p* > 0.05.

* *p* < .05.

Furthermore, the prevalence of genotype and allelic frequencies of the three SNPs in *TLR4* were examined with regards to smoking in patients with CVDs and healthy controls (Table 6). Only rs10759931 showed an increased risk for developing CVDs in patients that smoked. In patients with CVDs that smoked, the G allele frequency of rs10759931 increases the risk of developing CVDs by more than 1.5‐fold (OR = 1.557, 95% CI = 1.004–2.413, and *p* = .04694; Table 6A). Furthermore, the *TLR4* polymorphisms rs2770150 and rs4986790 did not show any correlation to CVDs with regards to smoking (Table 6A,B). After Bonferroni correction only patients without hypertension showed significant risk of CVD with rs2770150, other SNPs did not showed any association.

### 3.4 Comparison of the genotypes distribution for *TLR4* SNPs between KSA population and others populations

**Figure 1:**
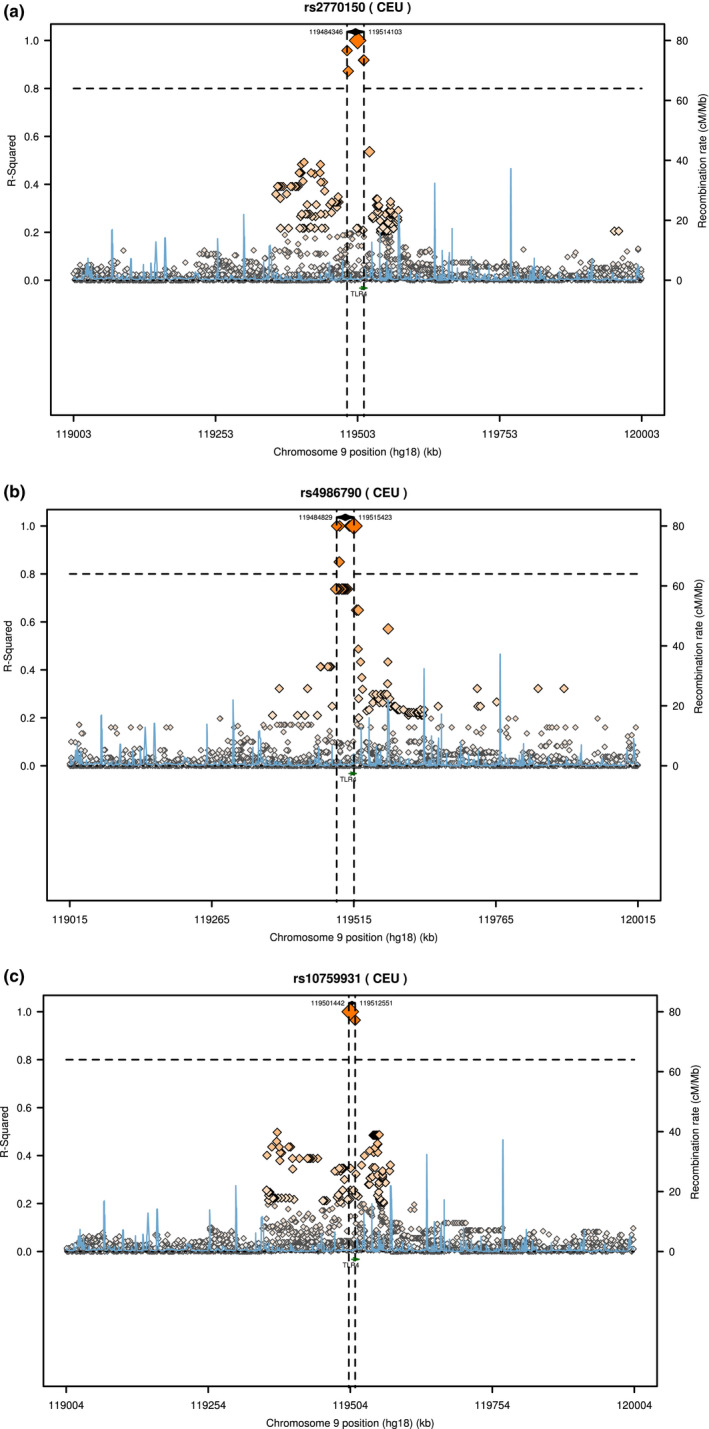
Regional LD plot for (a) *TLR4* rs2770150 SNP, (b) *TLR4* rs4986790 SNP, and (c) *TLR4* rs10759931 SNP. Abbreviations: CEU, Caucasian; cM, centiMorgan; kb, kilobase; LD, linkage disequilibrium; Mb, megabase; SNP, single‐nucleotide polymorphism; TLR, Toll‐like receptor

We have compared the studied genotypic distribution of three *TLR4* SNPs (rs2770150, rs4986790, and rs10759931) in Saudi Arabian control population to others population according to literature data available from the International HapMap project (http://hapmap.ncbi.nlm.nih.gov/). Our study showed that the genotypes frequency of *TLR4* rs2770150, was highly different with *p* < .05 between our study population (KSA) and the populations available from the HapMap project (Table7), suggest that, *TLR4* can to be used as specific marker for CVDs diagnostic in this ethnic. However, the genotype frequency of *TLR4* rs4986790 was similar in KSA population and European ancestry (CEU), Yoruba in Ibadan, Nigeria (YRI), African ancestry in Southwest USA (ASW), Gujarati Indians in Houston (GIH), Luhya in Webuye, Kenya (LWK), Mexican ancestry in Los Angeles (MEX), Maasai in Kinyawa, Kenya (MKK), and Toscani in Italia (TSI). However, this frequency differs for Han Chinese in Beijing, China (HCB), and Japanese in Tokyo (JPT; Table7; *p* < .05). No comparison was done for *TLR4* rs10759931 between KSA population and other population because the TLR‐4 SNP rs10759931 has no strong data available from the HapMap project, only low coverage panel data. Also, a comparison for regional linkage disequilibrium plot for *TLR4* SNPs was performed using SNPAnnotation and Proxy Search (http://www.broadinstitute.org/mpg/snap/ldplot.php) as shown in Figure 1.

**Table 7:** Comparison of TLR‐4 rs2770150 and rs4986790 SNPs genotypes distribution between KSA and others HapMap project populations

Population	Genotype Frequency			Allele Frequency		χ^2^	*p*‐value
	TT	CC	TC	T	C		
	*N* (%)	*N* (%)	*N* (%)	*N* (%)	*N* (%)		
TLR‐4 rs2770150							
CEU (226)	117 (0.52)	23 (0.1)	86 (0.38)	65 (0.28)	161 (0.71)	63.518	*<.05
HCB (86)	84 (0.98)	0 (0)	2 (0.02)	1 (0.01)	85 (0.99)	105.81	*<.05
JPT (88)	88 (1)	0 (0)	0 (0)	0 (0)	88 (1)	111.47	*<.05
YRI (226)	181 (0.8)	0 (0)	45 (0.2)	61 ( 0.27)	65 (0.73)	69.601	*<.05
CABG (217)	1,172 (0.5)	195 (0.09)	803 (0.37)	586 (0.27)	1584 (0.3)	138.32	*<.05
ASW (98)	76 (0.78)	0 (0)	22 (0.22)	11 (0.11)	87 (0.89)	83.11	<.05
CHB (82)	82 (1)	0 (0)	0 (0)	0 (0%)	82 (1)	105.89	*<.05
CHD (170)	168 (0.99)	0 (0)	2 (0.01)	2 (0.01)	168 (0.99)	172.53	*<.05
GIH (176)	97 (0.55)	12 (0.07)	67 (0.38)	48 ( 0.27)	128 (0.73)	60.371	*<.05
LWK (180)	135 (0.75)	4 (0.022)	40 (0.22)	24 (0.133)	157 (0.87)	113.46	*<.05
MEX (100)	64 (0.64)	10 (0.1)	26 (0.26)	23 (0.23)	77 (0.77)	52.944	*<.05
MKK (286)	206 (0.72)	14 (0.048)	66 (0.23)	46 (0.16)	240 (0.84)	131.81	*<.05
TSI (176)	110 (0.62)	8 (0.045)	58 (0.33)	35 (0.2)	141 (0.8)	85.150	*<.05
KSA (190)	88 (0.47)	81 (0.42)	20 (0.11)	129 (0.68)	61 (0.32)	Ref	

Population	Genotype frequency			Allele frequency		χ^ 2^	*p*‐value
	AA	GG	AG	A	G		
	*N* (%)	*N* (%)	*N* (%)	*N* (%)	*N* (%)		
TLR‐4 rs4986790							
CEU (226)	212 (0.94)	2 (0.01)	12 (0.05)	217 (0.96)	9 (0.04)	0.6104	.19420
HCB (90)	90 (1)	0 (0)	0 (0)	0 (0)	90 (1.0)	34.864	<.05*
JPT (90)	90 (1)	0 (0)	0 (0)	0 (0)	90 (1.0)	1.3388	<.05*
YRI (224)	207 (0.92)	0 (0)	18 (0.08)	213 (0.95)	11 (0.05)	1.0969	.40204
ASW (98)	88 (0.90)	2 (0.02)	8 (0.08)	93 (0.95)	5 (0.05)	11.625	.56325
GIH (174)	142 (0.816)	2 (0.011)	3 (0.017)	157 (0.90)	17 (0.1)	2.8201	.31024
LWK (176)	143 (0.81)	0 (0)	33 (0.19)	158 (0.90)	18 (0.1)	1.7322	.24518
MEX (98)	92 (0.94)	0 (0)	6 (0.06)	147 (0.97)	5 (0.03)	16.926	.14373
MKK (154)	130 (0.84)	1 (0.01)	23 (0.15)	313 (0.92)	27 (0.08)	2.924	.64597
TSI (284)	258 (0.91)	0 (0)	26 (0.09)	168 (0.95)	8 (0.05)	13.890	.34519
KSA	166 (0.87)	1 (0.01)	20 (0.11)	352 (0.92)	22 (0.06)	Ref	

Abbreviations: ASW, African ancestry in Southwest USA; CEU, European ancestry; CHD, Chinese in Metropolitan Denver, Colorado; GIH, Gujarati Indians in Houston; HCB, Han Chinese in Beijing, China; JPT, Japanese in Tokyo, Japan; KSA, population of Kingdome of Saudi Arabia; LWK, Luhya in Webuye, Kenya; MEX, Mexican ancestry in Los Angeles, California; MKK, Maasai in Kinyawa, Kenya;TLR, Toll‐like receptor; TSI, Toscani in Italia.; YRI, Yoruba in Ibadan, Nigeria.

* *p* < .05.

## 4 DISCUSSION

Cardiovascular diseases are immune inflammatory diseases. TLRs play an important role in the innate immune system by regulating inflammatory reactions and activating adaptive responses to eliminate harmful pathogens. A large number of SNPs have been discovered in the genes of these receptors, but the functional consequences in the majority of these SNPs have not yet been revealed. Some associations between TLRs polymorphisms and diseases have been previously reported (El‐Omar, Ng, & Hold, 2008) The development of cardiovascular diseases such as atherosclerosis, myocardial remodeling, valvular disease, and thrombosis occur as a result of the interactions between innate immunity and TLRs (Sharma, Garg, & Ashraf, 2016). Hence, this study is the first report to examine the possible associations between three *TLR4* polymorphisms with CVDs in the Saudi population. This will allow us to identify SNP biomarkers that are associated with CVDs, which can then be used to enhance the decision‐making of treatments for patients with CVDs. Our results clearly indicate that, of the three SNPs in *TLR4* that were analyzed, a significant association with CVDs was observed only for rs2770150, specifically in patients older than 60 years of age, in female patients, and in patients with CVDs that are not suffering from hypertension events. Furthermore, rs10759931 was also associated with an increased risk of developing CVDs in patients older than 60 years of age, in male patients, and in patients with CVDs that smoked. Finally, rs4986790 was not significantly associated with CVDs in the Saudi population. It is very important to clarify that our study subpopulations presents a significantly low frequency for TT genotype and G allele respectively for rs2770150 and 10,759,931 in the normal control subjects, compared to the CVD cases, implicates this genotype or allele can be considered as a risk factor in the Saudi population. However, for rs2770150, individuals with the TT genotype had a twofold higher risk for developing CVDs than those with the CC genotype. Furthermore, these SNPs are located in the regulatory region of *TLR4*. We hypothesize that rs2770150 and rs10759931 might affect the affinity of transcription factors binding to the regulatory region of *TLR4*, thus leading to deficiencies in TLR4 expression in patients with CVDs. An alternative hypothesis is that these SNPs affects the extracellular domain of the TLR4 receptor and is associated with a blunted response to TLR4 ligands such as LPS in humans (2000). Accumulating evidence also supports a role of dysregulated TLR signaling in the pathogenesis of infectious and autoimmune diseases including CVDs. Additionally, previous studies have shown that TLR4 expression was associated with CVDs with regards to left ventricular dysfunction. Alvas et al. propose that TLR4 expression may play a critical pathogenic role and may be used as an additional marker of ischemic myocardial dysfunction (Avlas et al., 2015). However, the regulation of TLR4 expression in cardiovascular diseases remains elusive. Lin, Chen, Lin, et al., 2006 and Lin, Chen, Tasi, et al., 2006 hypothesized that oxidative stress modify the posttranscriptional stabilization of TLR4 mRNA in vascular cells (Avlas et al., 2015; Lin, Chen, Lin, et al., 2006). TLR4 has also been reported to be found on cardiomyocytes cells with an increased secretion of proinflammatory cytokines such as tumor necrosis factor α (TNF‐α) and interleukin 1β (IL‐1β) through the nuclear factor‐κB (NFκB) pathway (Arslan, Kleijn, & Pasterkamp, 2011). Theses cytotoxic mediators are known by their role in myocyte damage (Fallach et al., 2010). Shao et al. showed that TLR4 can be used as a potential clinical biomarker for cardiovascular risk in CAD (Shao, Zhang, Zhang, Lu, & Ma, 2014). Several SNPs have been identified in the promoter and coding regions of TLRs, and their associations with infectious diseases as well as cardiovascular diseases are discussed below. The polymorphism rs352139, located in the intronic region of TLR4, has been demonstrated to be associated with the susceptibility to tuberculosis in Indonesian females (Kobayashi et al., 2012). Additionally, Lin et al. have shown that an Asp119Cys mutation in TLR4 increases the risk for ischemic stroke in Chinese population by 11.7‐fold (Lin et al., 2005). Although the role of TLR4 polymorphisms in CVDs have been inconsistent in the literature, recent reports have shown that the TLR4 rs4986790 polymorphism did not affect the risk of cardiovascular events in patients with rheumatoid arthritis (Chen, Gu, Gao, & Cen, 2015; Garcia‐Bermudez et al., 2012; Koch, Hoppmann, Pfeufer, Schomig, & Kastrati, 2006), which was consistent with our current results. The association of rs2770150 in CVDs has not been reported elsewhere, even though this SNP has been shown to be associated with other diseases such as gastric cancer (Santini et al., 2008) and prostate cancer (Chen et al., 2005) in other ethnic groups.

How the TT genotype for rs2770150 and the G allele for rs10759931 contribute to the susceptibility to CVDs based on gender in patients older than 60 years of age is not fully clear. Previous studies have suggested that significant genotype or allele differences exist in CVD incidences between males and females (Neyrolles & Quintana‐Murci, 2009; Zhao, Ying, Demei, & Xie, 2012). We hypothesize that variations in the expression of sex hormones and genetic variability between males and females might contribute to the differential incidence of infectious diseases as CVDs (Guerra‐Silveira & Abad‐Franch, 2013). Sex hormones play an essential role in the pathogenesis of CVDs, and a lower incidence and severity of CVD have been reported in women than men across all age groups. Estrogen has a protective role in the heart to prevent or delay CVDs (Vitale, Mendelsohn, & Rosano, 2009) and a decline in estrogen levels with age as well as menopause can lead to a rise of CVD incidence in women (Tan, Gast, & Schouw, 2010). Even though CVDs occur more frequently in men than women, both genders can inherit this disease. Therefore, further research should be performed to understand the factors that contribute to CVD in both men and women (Blauwet, 2011). In more than 30% of the healthy population, estrogen confers a protective effect protection against CVDs in women before menopause (Lerner & Kannel, 1986). Moreover, studies in vitro have revealed that progesterone regulates antiviral immunity through the inhibition of TLR‐mediated IFN‐alpha production (Hughes, Thomas, Li, Kaja, & Clark, 2008). In contrast, Semlali et al. showed that rs2770150 is associated with colon cancer in women older than 50 years of age and is closely linked with declining levels of female sex hormones during the postmenopausal period (Semlali et al., 2016). Furthermore, our data showed that the allele frequency of rs10759931 was significantly associated with CVD in male patients of the same age group, which is consistent with previous studies that investigated the involvement of this SNP with inflammatory diseases, including cancer (Singh, Singh, Agrawal, Gupta, & Singh, 2013; Song et al., 2009). Additionally, in contrast to our data, hypertension is the most common risk factor for susceptibility to cardiovascular diseases. The association between the selected SNPs in patients with CVDs and hypertensions was evaluated, showing that the TT genotype of rs2770150 in patients with CVDs without hypertension was almost three times higher when compared to the healthy controls. Therefore, we hypothesize that a suppressor mutation is present in the patients with CVDs and hypertension events.

Additionally, cigarette smoking is widely known to induce a higher risk for developing several chronic disorders including several types of cancer and chronic obstructive pulmonary diseases. Many studies have shown that cigarette smoking is a major cause of coronary heart disease, which can lead to heart attacks. In the current study, we found a significant association between rs10759931 and smoking in patients with CVDs. Moreover, the frequency of the G allele had an odds ratio greater than 1.5 in patients with CVDs that smoked when compared to healthy controls. Our results are in agreement with other previous studies that reported an association between *TLR4* polymorphism with related‐disease chronic obstructive pulmonary disease in smokers (Sabroe et al., 2004; Speletas et al., 2009).

*TLR4* polymorphisms play an influential role in CVDs. Our results indicate that rs2770150 is associated with female patients with CVDs over 50 years old without hypertension, whereas rs10759931 is strongly linked to male patients with CVDs over 50 years old that smoked. We propose that these SNPs can be utilized as biomarkers for the early detection of CVDs and for improving the decision‐making process when selecting treatments for CVDs.

## CONFLICT OF INTEREST

All authors have approved the manuscript and declare that there is no conflict of interest.
