# Supplementary material for: Toll‐like receptor 4 polymorphisms in Saudi population with cardiovascular diseases
Source: Mol Genet Genomic Med. 2019 Jul 21;7(9):e852. doi: 10.1002/mgg3.852 (PMC7650605; doi:10.1002/mgg3.852)
Supplement: Supplementary file 1 [file MGG3-7-e852-s001.docx]

**Supplementary Material Table 1**: Characteristics of selected polymorphisms involved in the Toll-like receptors 4

| SNP ID | Chr/ Position | Nucleotide  change | Region |
| --- | --- | --- | --- |
|  |  |  |  |
| **rs2770150** | **Chr 9**/ 117700861 | −3612T>C | 5'Upstream |
| **rs10759931** | **Chr9**/  117701869 | −2604G>A | Promoter |
| rs**4986790**  Asp299Gly | **Chr 9**/  117713024 | 8552A>G | Exon |
